# Males with low serum levels of vitamin D have lower pregnancy rates when ovulation induction and timed intercourse are used as a treatment for infertile couples: results from a pilot study

**DOI:** 10.1186/s12958-015-0126-9

**Published:** 2015-11-21

**Authors:** Massimo Tartagni, Maria Matteo, Domenico Baldini, Mario V. Tartagni, Hala Alrasheed, Maria A. De Salvia, Giuseppe Loverro, Monica Montagnani

**Affiliations:** Department of Biomedical Sciences and Human Oncology, School of Medicine, University of Bari “Aldo Moro”, Bari, Italy; University Department of Gynecology and Obstetrics - Ospedali Riuniti di Foggia, Viale Pinto, 71100 Foggia, Italy; Centro di Fecondazione Medicalmente Assistita MoMò Fertilife, Bisceglie, Italy

**Keywords:** Vitamin D, Hypovitaminosis D, Male infertility, Pregnancy rate

## Abstract

**Background:**

Vitamin D (Vit D) is important for the regulation of reproductive physiology. In humans, maternal Vit D deficiency has been implicated in several reproductive- and pregnancy-related disorders. Very few data are available regarding the Vit D status in male partners of couples attempting pregnancy. This observational study (IRB Prot. N. 078/13) aimed to evaluate whether low Vit D serum levels in males might decrease the rate of successful conception in couples attempting pregnancy.

**Methods:**

Male and female partners of infertile couples (*n* = 102) were classified into 2 GROUPS according to normal (≥30 ng/ml) or low (below 30 ng/ml) serum Vit D levels in male partners. Semen analysis was performed in each male participant based on the WHO reference criteria. The female partners of both groups were subjected to 3 consecutive cycles of gonadotropin-induced mono-ovulation. The main outcome measures included the clinical pregnancy rate, delivery per patient and per cycle, and miscarriage rate between the 2 groups evaluated at the end of the three-month period of the study.

**Results:**

In male partners of both groups, standard semen analysis did not highlight substantial differences in sperm concentration, sperm progressive motility, or typical form. The pregnancy rates per patient and per cycle and delivery rates per patient and per cycle were all significantly higher (*p*< 0.05) in couples with normal Vit D levels.

**Conclusions:**

These results suggest the existence of a relationship between male Vit D serum levels and semen ability to begin a pregnancy during cycles of timed vaginal intercourse.

## Background

The steroid hormone 25-hydroxycholecalciferol (Vitamin D (25(OH)D; Vit D)) has been recognized for its relevance to bone health and calcium homeostasis. In recent years, a number of experimental, observational and interventional studies have acknowledged the preventative role played by Vit D in physiological non-skeletal functions [1, 2]. Vit D deficiency (25(OH)D: <20 ng/mL) and insufficiency (25(OH)D: 20–29 ng/mL) are associated with skeletal diseases such as rickets, osteomalacia, osteoporosis and with chronic illnesses including autoimmune diseases such as multiple sclerosis or type 1 diabetes, inflammatory bowel disease such as Crohn’s disease, infections, especially of the upper respiratory tract, cardiovascular disturbances including hypertension, coronary artery disease, and heart failure, cancer such as colon and breast cancer, or non-Hodgkin’s lymphoma, and neurocognitive disorders, such as Alzheimer disease [3–6].

In addition to genetic predisposition [7], Vit D deficiency often results from inadequate dietary intake or insufficient cutaneous production [8]. In humans, the predominant source of Vit D is represented by the endogenous cutaneous synthesis, and dietary sources contribute to less than 20 % of the circulating levels of Vit D [9]. In the dermal fibroblasts and epidermal keratinocytes, the endogenous synthesis of Vit D occurs after photolytic conversion of 7-dehydrocholesterol (7(OH)D) by the ultraviolet B component of sunlight [10]. Subsequently, 7(OH)D is hydroxylated to 25(OH)D3 in the liver and is finally transformed to the active form of the vitamin, 1,25-dihydroxyvitamin D (1,25(OH)_2_D), in the kidney and in other target tissues [11, 12]. Active 1,25(OH)_2_D exerts genomic and non-genomic actions via its cognate nuclear receptors [13]. Typically, in subjects exposed to upwards of 300 h of sunshine per month, serum levels of 25(OH)D increase to the range of 35 ng/mL [14]. However, because the half-life of 25(OH)D is approximately 2–3 weeks [15], serum levels decline below the desired values of 30 ng/mL within 1–2 months when sunlight could no longer produce any vitamin D in the skin. This decline might be particularly relevant in the North European population. An ongoing clinical trial is evaluating the effects of Vit D supplementation in infertile Danish men [16]. In addition to latitude, several other factors including environmental pollution and increasing concerns about the carcinogenic potential of sunlight and subsequent increased use of sunscreen are recognized contributors to Vit D insufficiency [17], which has become a worldwide health issue [18–20]. Thus, even in a Mediterranean country, levels of Vit D might frequently decline below desired levels, especially during the winter [21].

The regulatory role of Vit D in reproductive physiology is highlighted by the expression of vitamin D receptors (VDR) and enzymes that metabolize Vit D in the ovary, uterus, and placenta and in the testis, male reproductive tract and human spermatozoa [22, 23]. The phenotype of the Tokio strain of mice with a disrupted VDR binding domain (*Vdr*^*−/−*^) is characterized by severely impaired fertility and histological abnormalities of the gonads from both sexes [24]. The importance of Vit D for male reproduction has been demonstrated in rodents by studies reporting that Vit D deficiency leads to reduced sperm counts, impaired sperm motility and lower fertility rates in females inseminated with semen from Vit D deficient males [25, 26]. In humans, the VDR and all the Vit D metabolizing enzymes are co-expressed during the late stages of spermatogenesis in the neck of mature spermatozoa [27]. It is likely that optimal sperm function might be influenced by Vit D directly or indirectly through Vit D-related calcium homeostasis [28]. Although a role for calcium in the maturation of human spermatozoa is supported by the 2–3-fold higher calcium concentration in the epididymal and prostate fluid compared with serum [29], the precise effects of Vit D on human sperm motility and vitality remains unclear. Based on the observations outlined above, it is plausible that Vit D deficiency might worsen the quality of sperm and significantly reduce its fertilizing capacity. The Vit D status in humans is measured by serum levels of 25(OH)D, and serum values below 30 ng/ml indicate Vit D insufficiency [30, 31].

This observational, pilot study was planned to evaluate whether Vit D levels might play a role on reproductive outcomes and whether low Vit D serum levels in males living in a Mediterranean country might decrease the rate of successful conception in couples attempting pregnancy.

## Methods

All procedures were in accordance with the Helsinki Declaration on Human Experimentation and were approved by the local Institutional Review Board (IRB) (Prot. N 078/13). One hundred and two (102) young couples reporting at least 1 year of sexual intercourse without the use of any contraceptive method were selected for the study between January 2014 and October 2014; all couples were accessing our academically affiliated private infertility center. Each eligible participant received a detailed explanation regarding the significance of an observational study evaluating the role of low/insufficient levels of Vit D in male partners on the rate of successful pregnancy. The subjects consenting to be enrolled and fulfilling the inclusion criteria were asked to sign a written informed consent. The study was carried out from November 2014 to January 2015.

In women, the ages ranged from 23 to 35 years (29.7 ± 2.6 years) and the infertility period ranged from 12 to 36 months (28.8 ± 3.5 months). All female patients had normal glucose tolerance according to the American Diabetes Association recommendations [32] and normal markers of thyroid, liver, and kidney function; none of the subjects were observing a specific diet or had been taking oral contraceptives or other long-term drugs in the 6 months preceding the beginning of the study. In all women, the endocrine profile was normal, according to values of the following hormones measured on day 3 of the cycle: for follicle-stimulating hormone (FSH) and luteinizing hormone (LH), the serum levels were <10 mUI/ml; prolactin levels were <15 ng/mL, and testosterone levels were <0.6 ng/mL. In all cases, progesterone (P) levels in the mid-luteal phase were higher than 6 ng/mL. All women had appropriate late luteal phase endometrial biopsies, normal cervical mucus, and bilateral tubal patency. The body mass index (weight/height^2^, expressed as kg/m^2^), plasma estrogen levels, and follicle diameter were recorded on day of human chorionic gonadotropin (hCG) administration. All hormone determinations were performed with appropriate commercial kits (Diagnostic System Laboratories Inc, Webster, Tex); intra-assay and inter-assay coefficients of variation were less than 10 %.

Serum Vit D levels were assessed in men and women by measuring serum 25(OH)D levels by a chemiluminescence immunoassay (Liaison Assay; Diasorin; Se: 4; CV%: 4.8 %–5.5 %). This assay accurately quantifies the sum of 25(OH)D3 and 25(OH)D2. Female patients whose Vit D levels were considered in the range of insufficiency (25(OH)D: 20–29 ng/ml) were given exogenous Vit D to obtain values higher than 30 ng/ml before the beginning of study, and their Vit D values were maintained in the control range throughout the study. Couples enrolled in this study were classified into 2 groups according to the Vit D values of the male partners. The male partners in couples of GROUP 1 - Normal VD (*n* = 39) had normal Vit D levels (≥30 ng/ml); the male partners in couples of GROUP 2 – Low VD (*n* = 63) had Vit D levels below 30 ng/ml. Couples were excluded if either the male or female partners were subjected to treatments interfering with Vit D metabolism. The male participants from both groups were advised not to change their diets during the period of the study. Men with diabetes mellitus, thyroid disease, or inflammatory bowel disease were not included in the study.

Among the male partner participants, seminal plasma from the ejaculate was obtained by voluntary masturbation after 3 days of sexual abstinence. Samples collected into a sterile container were allowed to liquefy for 30 min at room temperature before being analyzed according to standard World Health Organization (WHO) protocols [33, 34]. Standard semen analysis was performed by two experienced technicians operating under an active quality control program.

In both groups, the female partners were subjected to 3 consecutive cycles of gonadotropin-induced mono-ovulation under ultrasound monitoring, followed by follicular-timed vaginal intercourse. Three consecutive cycles of treatment were evaluated to prevent carryover effects of repeated ovarian stimulations [35]. Ovarian stimulation was performed with recombinant FSH (hrFSH, follitropin beta; Puregon, NV Organon, Oss, The Netherlands) starting at a daily dose of 50–100 IU on the third day of the cycle. Transvaginal ultrasound examinations were repeated every other day from the 5th day of treatment until the mean diameter of the dominant follicle measured 14 mm; thereafter, transvaginal ultrasounds were performed daily. Human chorionic gonadotropin (hCG; 10.000 IU; Gonasi IBSA, Rome, Italy) was administered when the mean diameter of the follicles reached 18 mm. Vaginal intercourse was suggested during the 30–36 h following hCG administration.

### Main outcome measures

The main outcome measures included the clinical pregnancy rate, delivery per patient and per cycle, and miscarriage rate between the 2 groups evaluated at the end of the three month period of the study. For the purpose of this study, the overall number of clinical pregnancies was calculated. Clinical pregnancy was defined as the presence of either an embryonic heart beat on transvaginal ultrasonography or the presence of a trophoblast at histologic evaluation after spontaneous abortion.

### Statistical analysis

Data were analyzed using Statistica software from StatSoft (Tulsa, Oklahoma, USA). Differences in baseline characteristics, clinical pregnancy rate, delivery per patient and per cycle, and miscarriage rate between the 2 groups were evaluated with Pearson’s *χ*^2^ test with Yates’ continuity correction and Fisher’s exact test. The results are expressed as the mean ± standard deviation (SD) unless otherwise specified. A value of *P* < 0.05 was considered to indicate statistical significance.

## Results

The 102 couples enrolled in this study were classified into 2 groups according to serum Vit D levels in male partners; 39 couples were assigned to GROUP 1 – Normal Vit D (≥30 ng/ml), and 63 couples were assigned to GROUP 2 – Low Vit D (<30 ng/ml). During the three month period of the study, 12 couples (3 in GROUP 1 and 9 in GROUP 2) failed to complete the 3 consecutive cycles of treatment and were excluded from the study. In total, 90 out of 102 couples (36 in GROUP 1 and 54 in GROUP 2) completed the study.

There were no substantial differences in the physiological parameters or biochemical markers of the male partners from the two groups (Table 1). Although the serum Vit D levels were significantly lower in the male patients of GROUP 2 compared with GROUP 1 (*P* < 0.001), no substantial difference was observed in the sperm concentration, sperm progressive motility, or typical form percent in samples from the subjects of GROUP 1 compared with subjects of GROUP 2 (Table 2).

**Table 1:** Characteristics of male partners in the 2 groups

	GROUP 1	GROUP 2	*P* Value
	Normal Vit D	Low Vit D	
Age (years)	34.1 ± 3.8	36.5 ± 1.4	n.s.
Body mass index (kg/m^2^)	24.6 ± 2.5	23.8 ± 2.7	n.s.
Systolic Blood Pressure (mmHg)	125.8 ± 11.3	121.2 ± 9.2	n.s.
Blood glucose (mg/dl)	101.7 ± 7.1	98.8 ± 10.7	n.s.
Serum PSA (ng/mL)^a^	2.6 ± 0.5	2.1 ± 0.6	n.s.
Smoking (n/tot)	12/36	17/54	n.s.

Values are mean ± SD

^a^ *PSA* prostate specific antigen

**Table 2:** Standard characteristics of semen in the 2 groups^a^

	GROUP 1	GROUP 2	*P* Value
	Normal Vit D	Low Vit D	
Serum Vit. D (ng/ml)	34.2 ± 3.38	18.4 ± 5.82	*p* < 0.001
Sperm concentration (x 10^6^/mL)	16.9 ± 2.41	16.1 ± 2.40	n.s.
Sperm progressive motility (%)	40.3 ± 2.99	39.7 ± 3.4	n.s.
Typical forms (%)	5.03 ± 0.99	5.11 ± 0.94	n.s.

^a^Values are mean ± SD

The main characteristics of the female partners in GROUP 1 and GROUP 2 are summarized in Table 3. The age, duration of infertility, BMI, plasma estrogen level, and follicle diameter on hCG day of administration were similar in women from both groups. Among the 90 couples recruited, 108 cycles of timed vaginal intercourse were performed in GROUP 1, and 162 cycles were performed in GROUP 2. Differences in clinical pregnancies, pregnancy rates per patient and per cycle, delivery rates per patient and per cycle, numbers of miscarriages and miscarriage rates are reported in Table 4. All parameters evaluating pregnancy and delivery were significantly higher in couples with normal serum levels of Vit D (GROUP 1) than in couples with low Vit D levels (GROUP 2). A tendency towards a higher rate of miscarriage was observed in subjects of GROUP 2; although the miscarriage rate was higher in GROUP 2 than in GROUP 1, this parameter did not reach statistical significance (*p*> 0.23). No hyperstimulation or ectopic pregnancies occurred in either group.

**Table 3:** Characteristics of female partners in the 2 groups^a^

	GROUP 1	GROUP 2	*P* Value
	Normal Vit D	Low Vit D	
Number of Couples	36	54	n.s.
Age (years)	30.1 ± 2.57	29.5 ± 2.57	n.s.
Duration of infertility (months)	28.5 ± 4.53	29.1 ± 2.57	n.s.
Serum estradiol (pg/mL)^b^	291.2 ± 31.73	293.1 ± 19.81	n.s.
Follicle diameter (mm)	18.2 ± 1.28	18.0 ± 1.34	n.s.
Body mass index (kg/m^2^)	20.8 ± 1.14	21.1 ± 1.37	n.s.

^a^Values are mean ± SD

^b^On the day of hCG administration. Conversion factor to SI unit = 3.671

**Table 4:** Number of total cycles and pregnancy rate per patient and per cycle in the 2 groups^a^

	GROUP 1	GROUP 2	*P* Value
	Normal Vit D	Low Vit D	
No. of couples	36	54	n.s.
No. of cycles	108	162	n.s.
No. of clinical pregnancies	10	5	n.s.
No. of term pregnancies	8	2	n.s.
Pregnancy rate/patient (%)	27.7	9.2	*p* < 0.05
Pregnancy rate/cycle (%)	9.2	3.0	*p* < 0.05
Delivery rate/patient (%)	22.2	3.7	*p* < 0.02
Delivery rate/cycle (%)	7.4	1.2	*p* < 0.01
No. of miscarriage	2	3	n.s.
Miscarriage rate (%)	20	60	n.s.

^a^ Values are mean ± SD

## Discussion

Human infertility is defined as the failure to achieve a clinical pregnancy after twelve months of regular, contraceptive-free sexual intercourse [36]. All couples recruited in this study did not attain pregnancy despite the lack of use of any contraceptive method during the last year of sexual intercourse. Twelve months is the lower reference limit for Time To Pregnancy (TTP), and TTP is a good indicator of fertility potential because it depends on regular ovulation, oocyte quality, and semen quality variables [37].

To our knowledge, this is the first study evaluating the correlation between serum Vit D levels in men and pregnancy rates in their female partners undergoing mono-ovulation induction and timed intercourse. The existing literature supports a solid role of Vit D for the regulation of reproductive physiology in male and female animal models [23, 38]. In humans, maternal Vit D deficiency has been implicated in several reproductive- and pregnancy-related disorders, ranging from PCOS to premature birth [39, 40]. Very few data are available regarding the Vit D status in male partners of couples attempting pregnancy [41].

To investigate whether male hypovitaminosis D might be a discriminating factor for successful pregnancy, the female partners of all couples in this study underwent identical procedures. The beneficial effects of ovulation induction in terms of improved oocyte quality, improved timing of the ovulatory period, and correction of slight and unknown ovulatory disorders have long been demonstrated [42, 43]. In female partners, we adopted a stimulation protocol with low doses of gonadotropin to obtain a cohort of mono-ovulatory patients that was as homogeneous as possible; in both groups, no cases of multiple pregnancies or hyperstimulation were observed. In the women of GROUP 1 and GROUP 2, the Vit D levels were in the control range (>30 ng/ml) for the duration of the study. Furthermore, the female patients assigned to GROUP 1 or GROUP 2 had comparable age distributions, estrogen plasma levels, numbers of rFSH ampoules used, and days of therapy (data not shown). Therefore, it is reasonable to presume that the oocytes in the women of the two groups were of comparable quality.

In the male partners of GROUP 1 and GROUP 2, standard semen analysis did not highlight substantial differences in sperm concentration, sperm progressive motility, or typical form. This observation might have several explanations. First, routine semen analysis itself has its own limitations, and does not account for putative sperm dysfunctions such as immature chromatin or fragmented DNA. In addition, semen components might be influenced by several other factors, such as sexual activity and accessory sex gland function. If anything, this finding confirms the poor predictive significance of semen analysis in terms of fertility potential.

Because the pregnancy rates per patient and per cycle were appreciably higher in couples of GROUP 1 (male partners with normal Vit D serum levels), the assumption that the worse prognosis of successful pregnancy is in couples whose male partners have low Vit D levels appears clear.

The results obtained in this study are in agreement with data supporting the relevance of Vit D in the regulation of male reproductive physiology. For example, the VDR has been shown to be present in the hypothalamic–pituitary axis, the testis and on the head/nucleus of the sperm and mid-piece [23, 44]. Although the exact role of the VDR in the sperm nucleus is unclear, it has been suggested to acts as a protective genomic factor because it is important for the proper control of sperm DNA integrity and the maintenance of genome stability [22].

Enzymes responsible for active Vit D conversion have been found in male reproductive tissues, and locally produced Vit D appears to modulate cholesterol efflux in human sperm, affect tyrosine and threonine phosphorylation of sperm proteins and increase sperm viability [45]. By increasing intracellular calcium concentrations, sperm motility and acrosin activity within the female reproductive tract, tissue-specific production of Vit D might significantly contribute to overall fertilizing capacity. The increased embryonic loss observed in couples whose male partners had low serum Vit D levels is consistent with this hypothesis.

Some limitations should be taken into account when considering the results. First, the small sample size of this observational, pilot study might not accurately address the contributions of other potential confounding factors to involuntary male infertility. However, the physiological and biochemical parameters related to the most well-known variables involved in male fertility (BMI, blood pressure, smoking habits, blood glucose profile) did not differ between the men of the two groups. Second, this study was carried out during the winter months, when Vit D levels are known to be the lowest. This period was purposely selected to stress the potential relevance of male Vit D insufficiency to fertilizing capacity in a Mediterranean population. Nevertheless, seasonal differences might be relevant to serum levels of Vit D, and this aspect should be adequately considered in further studies. Third, our study does not directly address the specific mechanisms by which Vit D is involved in reproduction, although some hypotheses could be suggested. For example, it is possible that insufficient Vit D levels in males might decrease protection from inflammatory signaling and oxidative stress in spermatozoa, as demonstrated in several other cell types [46–48]. This insufficiency might affect the functional integrity of the spermatozoa, therefore reducing sperm fertility potential by causing implantation disorders, rather than fertilization problems. Supernumerary defective spermatozoa could damage the oocyte or pro-nucleate embryo by altering the physical-chemical properties of the *zona pellucida* via the release of toxic metabolites such as oxygen radicals [49]. Subtle abnormalities in defective Vit D spermatozoa might lead to subsequent abnormal membrane function in the embryo and anomalies in the cell-to-cell communication/binding that appear to have an important role in the attachment of the blastocyst and penetration of surface epithelium of the endometrium [50]. Further support for this hypothesis comes from human studies indicating that paternally derived proteins are expressed in the embryo at the pre-implantation stage [51]. An additional mechanism that might help explain the relationship between sperm abnormalities and poor pregnancy outcomes is a desynchronization between slower embryonic development and the uterine environment [52]. According to this view, it is plausible that defective spermatozoa could lead to a less viable embryo, and therefore to a low viable pregnancy rate with a high miscarriage rate. Consistent with this hypothesis, a tendency towards a higher rate of miscarriage was observed in the couples of GROUP 2 in our study.

## Conclusions

Taken together, the results from our pilot study suggest the existence of a direct relationship between male Vit D serum levels and semen ability to begin a pregnancy during cycles of timed vaginal intercourse. Further studies are required to elucidate the pathophysiology of this condition and its relationship to overall male fertility. Given its recognized safety, accessibility and ease of administration, the evaluation of Vit D levels and subsequent Vit D supplementation in male individuals with serum levels below the normal range could represent a cost effective strategy to improve the chances of clinical pregnancy for couples undergoing mono-ovulation induction and timed intercourse.
